# Fornix Stimulation Induces Metabolic Activity and Dopaminergic Response in the Nucleus Accumbens

**DOI:** 10.3389/fnins.2019.01109

**Published:** 2019-10-24

**Authors:** Hojin Shin, Sang-Yoon Lee, Hyun-U Cho, Yoonbae Oh, In Young Kim, Kendall H. Lee, Dong Pyo Jang, Hoon-Ki Min

**Affiliations:** ^1^Graduate School of Biomedical Science & Engineering, Hanyang University, Seoul, South Korea; ^2^Department of Neuroscience, College of Medicine, Gachon University, Incheon, South Korea; ^3^Department of Neurologic Surgery, Mayo Clinic, Rochester, MN, United States; ^4^Department of Biomedical Engineering, Hanyang University, Seoul, South Korea; ^5^Department of Physiology and Biomedical Engineering, Mayo Clinic, Rochester, MN, United States; ^6^Department of Radiology, Mayo Clinic, Rochester, MN, United States

**Keywords:** fornix, nucleus accumbens, deep brain stimulation, dopamine, positron emission tomography, fast-scan cyclic voltammetry

## Abstract

The Papez circuit, including the fornix white matter bundle, is a well-known neural network that is involved in multiple limbic functions such as memory and emotional expression. We previously reported a large-animal study of deep brain stimulation (DBS) in the fornix that found stimulation-induced hemodynamic responses in both the medial limbic and corticolimbic circuits on functional resonance imaging (fMRI) and evoked dopamine responses in the nucleus accumbens (NAc), as measured by fast-scan cyclic voltammetry (FSCV). The effects of DBS on the fornix are challenging to analyze, given its structural complexity and connection to multiple neuronal networks. In this study, we extend our earlier work to a rodent model wherein we characterize regional brain activity changes resulting from fornix stimulation using fludeoxyglucose (^18^F-FDG) micro positron emission tomography (PET) and monitor neurochemical changes using FSCV with pharmacological confirmation. Both global functional changes and local changes were measured in a rodent model of fornix DBS. Functional brain activity was measured by micro-PET, and the neurochemical changes in local areas were monitored by FSCV. Micro-PET images revealed increased glucose metabolism within the medial limbic and corticolimbic circuits. Neurotransmitter efflux induced by fornix DBS was monitored at NAc by FSCV and identified by specific neurotransmitter reuptake inhibitors. We found a significant increase in the metabolic activity in several key regions of the medial limbic circuits and dopamine efflux in the NAc following fornix stimulation. These results suggest that electrical stimulation of the fornix modulates the activity of brain memory circuits, including the hippocampus and NAc within the dopaminergic pathway.

## Introduction

The Papez circuit is a well-known neural network that is involved in multiple limbic functions such as memory and emotional expression (Rajmohan and Mohandas, 2007). The circuit consists of the hippocampus, fornix, mammillary body, anterior nucleus of the thalamus, cingulate cortex, parahippocampal gyrus, and entorhinal cortex (Mesulam, 2000). Studies show that the interactions between the Papez circuit (medial limbic) and mesocorticolimbic circuits, including the amygdala (AM), nucleus accumbens (NAc), and prefrontal cortex (PFC) are vital to consolidation and retrieval of memory (Hamann et al., 1999; Lisman and Grace, 2005; Carr et al., 2011; Badre et al., 2014; Schedlbauer et al., 2014; Horner et al., 2015). Also, in dementia patients, memory decline has been shown to correlate with the dysfunction of intrinsic connectivity between the hippocampus, PFC, and striatum (Greicius et al., 2004; Buckner et al., 2005; Zhou and Seeley, 2014). These findings highlight the importance of studying the interaction between the medial limbic and mesocorticolimbic circuits (Ito et al., 2008; Mikell et al., 2009; Kahn and Shohamy, 2013; Bagot et al., 2015).

The fornix serves as the major structure between the hippocampus and the mammillary bodies with additional projections to the hypothalamus. Animal studies have demonstrated that lesions or transection of the fornix will hinder memory function in experimental behavior tests (Charles et al., 2004; Fletcher et al., 2006; Mala et al., 2012). A clinical trial investigating deep brain stimulation (DBS) of limbic circuitry as a treatment for morbid obesity resulted in unexpected effects on specific memory functions, evoking detailed autobiographical memories, and enhancing performance on associative memory tasks (Hamani et al., 2008). Based on this finding, DBS was applied to the fornix to address the memory dysfunction associated with dementia in a small cohort of patients with Alzheimer’s disease (AD) (Laxton et al., 2010; Fontaine et al., 2013; Holroyd et al., 2015; Lozano et al., 2016; Ponce et al., 2016). Although the results of the clinical trial were favorable, we do not fully understand the mechanism by which electrically stimulating the fornix bundle affects memory function, nor its broader effects on other circuits with which the fornix bundle interacts (Vann, 2013; Ross et al., 2016).

The major challenge in determining the effects of fornix DBS is the complexity of the neuroanatomic and functional connections of the fornix. It is involved in numerous cognitive processes that have diverse axonal pathways, such as the medial limbic and the mesocorticolimbic circuit components. Adding to the complexity is a wide range of axonal fiber effects involving the hippocampus, medial temporal lobe, and NAc (Saint Marie et al., 2010; Kahn and Shohamy, 2013; Bagot et al., 2015). In a large-animal functional magnetic resonance imaging (fMRI) study, we previously demonstrated that fornix DBS elicits functional interactions between the medial limbic and mesocorticolimbic circuits and partially regulates major excitatory input into and through the NAc (Ross et al., 2016).

There have been few systematic studies of the functional network effects of fornix stimulation or the neurochemical changes that it can induce. The goal of the present study was to characterize both the regional brain activity and the specific neurochemical changes associated with fornix stimulation in rodents. To do so, we used micro positron emission tomography (PET) imaging to measure the uptake and accumulation of fludeoxyglucose (^18^F-FDG) as a tracer for brain activity. Molecular imaging systems with specific tracers have been proposed as a biomarker-based approach to investigating the neural mechanisms for neurodegenerative diseases such as Parkinson’s disease (PD), AD, and epilepsy (Lenkov et al., 2013; Joutsa et al., 2017; Matarazzo et al., 2018; Valotassiou et al., 2018). This technique was developed to monitor specific regional brain activity in small animals (Mirrione et al., 2007; Jang et al., 2009a, 2012; Frankemolle et al., 2010). To monitor specific neurotransmitter changes, we used fast-scan cyclic voltammetry (FSCV), which is a real-time electrochemical monitoring system (Agnesi et al., 2009; Bledsoe et al., 2009).

## Materials and Methods

### Subjects

All procedures were performed in accordance with the National Institutes of Health Guidelines for Animal Research (Guide for the Care and Use of Laboratory Animals), and the Hanyang University Institutional Animal Care and Use Committee approved all experimental procedures. The subjects consisted of adult male Sprague Dawley rats (300–350 g, Koatech, Korea) (total *n* = 18; *n* = 15 for the PET study, and *n* = 3 for the FSCV study). Subjects were housed in cages containing two or three animals each, with 12-h light and dark cycles, 50–60% humidity, and *ad libitum* access to food and water.

### Stereotactic Surgery

**FIGURE 1:**
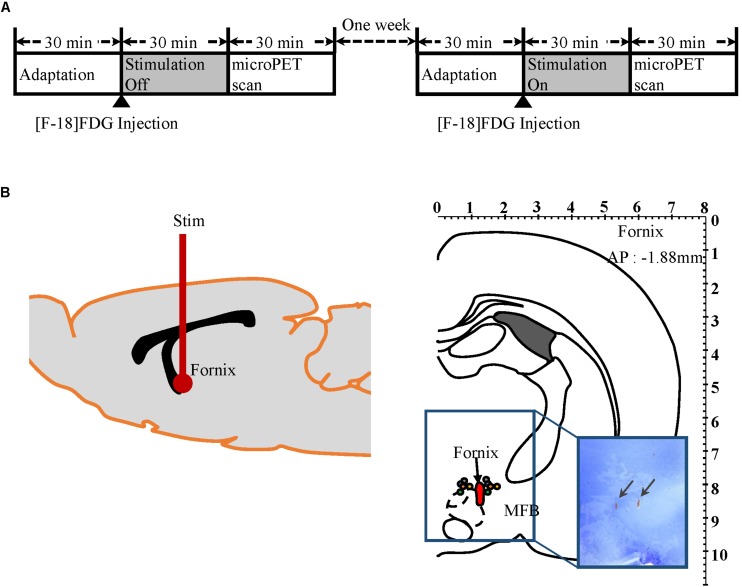
Micro-PET experiment protocol and electrodes placement verification by histology. **(A)** Mirco-PET experiment protocol diagram. **(B)** Left: Diagram of electrode location in rat brain. Right: Location of stimulation electrodes implanted into the fornix (AP −1.88 mm, Paxinos and Watson). Each pair of colored circles marks the tip of the stimulation electrode. Arrows in rectangular image indicate where electrode tips are located in brain histology slices.

All subjects were anesthetized with Zoletil (0.1 mL/100 g, 5 mg/mL, Virbac, France) 30 min before surgery and were positioned into a stereotaxic frame (David Kopf Instruments, United States). Body temperature was maintained at 37°C with a heating pad (TCAT-2, Harvard Apparatus, United States). A burr hole was drilled into the skull according to coordinates based on Paxinos and Watson’s Rat Brain Atlas (Paxinos and Watson, 1997). A twisted bipolar stainless steel electrical stimulation electrode (diameter 125 μm, 500 μm exposure, Polyimide coated, Plastics One, United States) was implanted into the fornix (AP: −1.88 mm, ML: +1.3 mm, DV: −8.3 mm) (Figure 1B). Once the stimulating electrode was positioned at the optimal location for targeting the fornix, it was fixed firmly to the skull with three to four screws that were fixed in place with light curing dental cement (CharmmFil Flow, DenKist, Korea). Subjects were monitored for a week of recovery post-surgery.

### Micro-PET Acquisition

Two micro-PET (Focus 120 MicroPET, Concorde Microsystems, Knoxville, TN, United States) scans were conducted for each of 15 subjects with either “stimulation on” or “stimulation off,” as outlined in Figure 1A. The 15 subjects were randomly assigned to one of two groups: “stimulation on” (*n* = 8) or “stimulation off” (*n* = 7) in the first week. In week 2, the conditions were reversed so that the eight subjects who had been scanned during “stimulation on” in the first week were scanned with “stimulation off,” and the seven subjects who had “stimulation off” in week 1 were scanned with “stimulation on” in week 2. Prior to micro-PET scanning, subjects were kept in cages for 30 min in a room maintained at 30°C to maximize ^18^F-FDG uptake, as previously described (Fueger et al., 2006). ^18^F-FDG (500 μCi/100 g) was injected into the tail vein under light anesthesia with Zoletil (0.03 mL/100 g, 5 mg/mL, Virbac, France). Subjects then stayed in an awake state in the cage for another 30 min for ^18^F-FDG uptake with or without electrical stimulation (100 μA amplitude pulses at 120 Hz and a pulse width of 2 ms, biphasic). After 30 min of uptake, subjects were put into the PET scanner. The transition time took <1 min. During the PET scan, subjects were under 2% isoflurane anesthesia.

### PET Post-processing and Statistical Analysis

**FIGURE 2:**
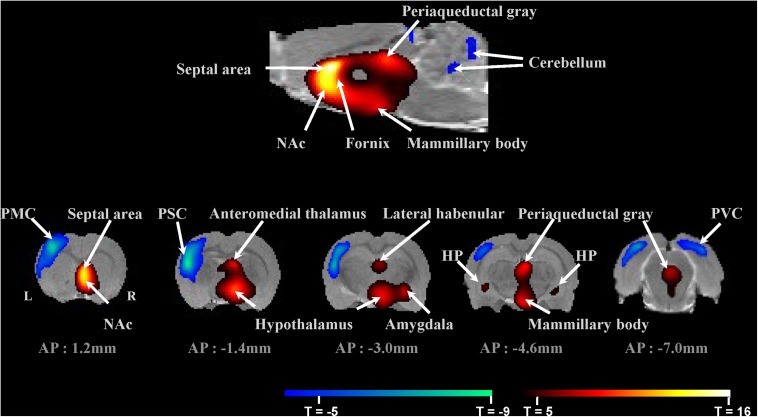
Micro-PET imaging of fornix stimulation-induced brain regional activity averaged across 15 subjects. Brain areas with significant changes in glucose metabolism induced by fornix electrical stimulation (FDR < 0.05, *n* = 15). **Top:** Mirco-PET image of sagittal section. **Bottom:** Micro-PET images of coronal sections through the rat brain from AP 1.2 mm to AP –7.0 mm. PMC, primary motor cortex; PSC, primary somatosensory cortex; PVC, primary visual cortex; NAc, nucleus accumbens; and HP, hippocampus.

**FIGURE 3:**
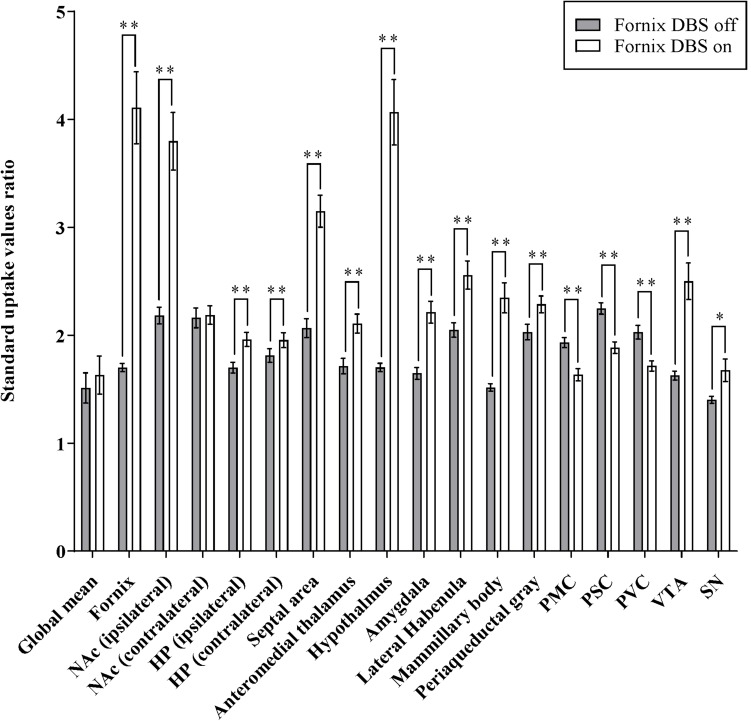
Effects of fornix deep brain stimulation on glucose metabolism. Comparisons of ^18^F-FDG uptake in the brain regions shown as increase/decrease relative to fornix stimulation “on” and fornix stimulation “off.” Data presented are mean ± SEM values. ^∗^Values that differ significantly between fornix DBS off and on according to paired *t*-test (^∗^*p* < 0.05, ^∗∗^*p* < 0.001). NAc, nucleus accumbens; HP, hippocampus; PMC, primary motor cortex; PSC, primary somatosensory cortex; PVC, primary visual cortex; VTA, ventral segmental area; and SN, substantia nigra.

Fludeoxyglucose micro-PET images were reconstructed using the ordered subset expectation maximization (OSEM) algorithm with 10 iterations. Using MRIcro (MRIcro Software, Georgia Institute of Technology, Atlanta, GA, United States), an individual mask was applied to extract the whole brain only. These images were normalized to an ^18^F-FDG rat brain template (Ref, Jang) using a statistical parametric mapping (SPM) program applying six-affine rigid-body transformation smoothed with an isotropic Gaussian kernel (1 mm FWHM). Proportional signal scaling was applied for global normalization. A general linear model was applied, conducting paired *t*-tests comparing stimulation on–off effects across subjects (*n* = 15) in SPM. The statistical threshold was set at *P* < 0.05 (false discovery rate, FDR). The ^18^F-FDG rat brain template has a matching T1-weighted magnetic resonance imaging (MRI) template that matches to Paxinos-atlas space (Schweinhardt et al., 2003; Jang et al., 2009b). The *t*-value map was overlaid onto the T1 MRI for display in Figure 2. The maximum *t*-value coordinate for each brain region was extracted based on the MRI template and Paxinos atlas. From these maximum *t*-value coordinates, individual raw FDG PET data were further extracted and normalized to the individual cerebellum value creating standard uptake values ratio (SUVR) used for Figure 3.

### Fast-Scan Cyclic Voltammetry

Three subjects that were not part of the micro-PET study underwent FSCV recording in NAc during fornix stimulation. Electrochemical changes were recorded using conventional electrochemical sensing carbon fiber microelectrodes (CFMs) fabricated as described previously (7 μm diameter; 50–100 μm length exposed) (Chang et al., 2012). All procedures were conducted under anesthesia with Zoletil (0.1 mL/100 g, 5 mg/mL, Virbac, France). Surgical procedures similar to those for inserting the stimulating electrode were followed when two additional burr holes were made for (1) CFM implantation into the NAc (AP: +1.2 mm, ML: +1.4 mm, DV: −6.5 mm to −7.8 mm) and (2) a reference electrode (Ag/AgCl) implanted into the contralateral hemisphere. Neurotransmitter changes in the NAc during fornix stimulation were measured using the Wireless Instantaneous Neurotransmitter Concentration Sensor (WINCS) system (Agnesi et al., 2009; Bledsoe et al., 2009). Conventional triangular waveforms (−0.4 to 1.5 V versus Ag/AgCl at 400 V/s) were applied at 10 Hz. Background subtraction was performed by subtracting the average of 10 voltammograms acquired prior to electrical stimulation from each voltammogram acquired after stimulation. FSCV recording began after the stabilization of the electrode. Once the stimulation electrode was fixed at the fornix, the dopamine release by electrical stimulation was measured by lowering the CFM 100 μm into the NAc each time, with 10 min interval for recovery. Electrical stimulation was applied by an isolated pulse stimulator system utilizing the parameters: 300 μA amplitude pulses at 120 Hz and a pulse width of 2 ms, biphasic, for 2 s, additional details are available in the Supplementary Figure S1 (A–M system Model 2100, United States). A pharmacological confirmation of NAc neurochemical changes due to fornix electrical stimulation was conducted in all three FSCV subjects (Lee et al., 2006). A dopamine selective reuptake inhibitor was administered after neurotransmitter detection. Nomifensine was obtained from Sigma–Aldrich (20 mg/kg, St. Louis, MO, United States) and dissolved in 0.9% NaCl saline and injected into the intraperitoneal space.

### Histology and Staining

At the end of the experiment after euthanization, target site confirmation was conducted by histology in five subjects randomly selected from the micro-PET experimental group to confirm the fornix site and in the three FSCV subjects to confirm the NAc site. Each subject was exposed to a high current (1 mA for 10 s) to mark electrolytic lesions with the electrodes. The brains were removed and stored for 24 h in 4% paraformaldehyde solution [40 g/L in phosphate-buffered saline (PBS)] at 4°C. The brains were then immersed in a 30% sucrose in PBS solution for 48 h until they sank to the bottom of the container. The brains were then sliced into 50-μm-thick sections and mounted on glass slides. Brain slices were placed directly into chloroform for 30 min and then rehydrated by applying decreasing concentrations (100, 95, and 70%) of ethyl alcohol in distilled water. Brain slices were stained with 0.1% cresyl violet solution and examined microscopically to determine the location of each electrode tip in the brain.

## Results

### Histological Verification of Electrode Positions

**FIGURE 4:**
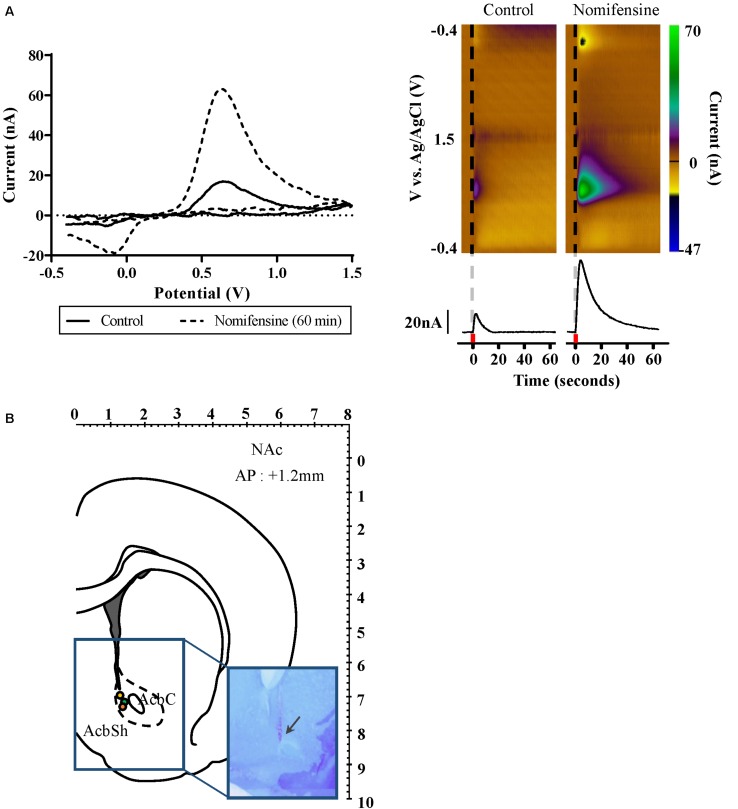
Dopamine release as measured by FSCV in the nucleus accumbens during fornix stimulation. **(A)** Left: Confirmation of dopamine release by using dopamine selective reuptake inhibitor; control (solid), and 60 min after dopamine reuptake inhibitor administered (dash). Background subtracted voltammogram from 60 min after dopamine reuptake inhibitor administered showed significant increase compared to control. Right: Representative color plots of dopamine *in vivo*. Black vertical line (dash) denotes time of fornix stimulation. Current versus time plot at +0.6 V comparing DA release before and after dopamine reuptake inhibitor administered. Red square indicates 2 s when fornix stimulation was applied. **(B)** Location of FSCV CFM electrodes implanted into the NAc (AP 1.2 mm, Paxinos and Watson). Each colored circle marks the tip of the FSCV CFM electrodes. Arrow in rectangular image indicates where CFM tip is located in the brain histology slices. FSCV, fast-scan cyclic voltammetry; CFM, carbon fiber microelectrode; and NAc, nucleus accumbens.

After the completion of the measurements, the positions of the electrodes for the micro-PET study were histologically verified (*n* = 5, Figure 1B). The histological analysis showed that the twisted bipolar stainless steel electrical stimulation electrodes had been implanted in the fornix area accurately. In targeting the fornix, we avoided the medial forebrain bundle (mfb), which contains direct dopaminergic fibers from the ventral tegmental area (VTA), and histology confirmed that the stimulation electrode was not in close proximity to the mfb. Histological analysis (Figure 4B) for the FSCV experiment showed that the CFM had been implanted accurately close to the NAc in all three FSCV subjects.

### Fornix Electrical Stimulation Results in Micro-PET Images Show Increased Glucose Metabolism Within the Medial Limbic and Corticolimbic Circuits

Figure 3 summarizes glucose metabolism changes during fornix electrical stimulation and their relative statistical significance, as determined by paired *t*-tests comparing “stimulation on” with “stimulation off.” Micro-PET imaging revealed that fornix stimulation induced a significant glucose metabolism increase in the medial limbic and corticolimbic circuits, including the hippocampus, mammillary body, and anteromedial thalamus (FDR < 0.05, *n* = 15) (Figure 2). Fornix stimulation also generated a robust glucose metabolism increase in the ipsilateral NAc as well as increases in numerous other regions, including the lateral habenular, periaqueductal gray, AM, and septal area. We also observed significant decreases in glucose metabolism in several regions, including the primary motor cortex (PMC), primary somatosensory cortex (PSC), primary visual cortex (PVC), and cerebellum. The global mean of FDG uptake revealed no significant differences between the fornix electrical stimulation on and stimulation off conditions. However, specific brain regions, including the fornix, NAc (ipsilateral), hippocampus, septal area, anteromedial thalamus, hypothalamus, AM, lateral habenula, mammillary body, periaqueductal gray, PMC, PSC, PVC, VTA, and substantia nigra (SN) did reveal significant differences in FDG uptake between the stimulation on and stimulation off conditions (^∗^*p* < 0.05, ^∗∗^*p* < 0.001). There were no significant differences in the contralateral NAc relative to the stimulation on and stimulation off conditions.

### Neurotransmitter Efflux Induced by Fornix Electrical Stimulation

We used FSCV to measure neurochemical changes in the NAc during fornix stimulation (*n* = 3). During phasic fornix stimulation (2 s), dopamine release was detected *in vivo* in the NAc (Figure 4). The current–voltage curve confirms the oxidation peak at 0.6 V and the reduction peak at −0.2 V, showing characteristic FSCV dopamine markers. For further confirmation, we administered nomifensine, an inhibitor specific to dopamine reuptake. As shown in Figure 4A, the dopamine reuptake inhibitor showed a significant increase of neurotransmitter efflux 60 min after administration compared to the control condition by fornix stimulation alone. As shown in Figure 4B, histologic analysis confirmed that the CFM had been accurately implanted close to the NAc in all three FSCV subjects.

## Discussion

In the present study, we confirmed that fornix stimulation within the Papez circuit could induce NAc activity and further efflux of dopamine. These findings are consistent with our large-animal fMRI and FSCV study of fornix stimulation (Ross et al., 2016) and a relevant recent rat study that showed that chronic forniceal DBS significantly reduces amyloid deposition in the hippocampus and cortex, decreases astrogliosis and microglia activation, and lowers neuronal loss (Leplus et al., 2019). The micro-PET results revealed that fornix stimulation increases glucose metabolism in medial limbic circuits, including the hippocampus, mammillary bodies, and anteromedial thalamus. Other regions, such as the septal area, lateral habenula, AM, and periaqueductal gray, also showed increased glucose metabolism. In contrast, glucose metabolism was decreased in the PMC, PSC, and PVC. These increases and decreases in brain activity suggest that the fornix is part of a major limbic system pathway, the Papez circuit, which is primarily involved in certain aspects of cortical control of emotional processing and memory storage.

Additionally, fornix stimulation significantly increased activation in the NAc. In particular, encoding and consolidation of memories require the stimulation of dopamine receptors as part of a hippocampal–striatal–prefrontal loop that orchestrates the formation of new memories (Lisman and Grace, 2005; Axmacher et al., 2010). This finding may reflect the strategic position of the fornix in the brain, in that ventral hippocampal glutamatergic afferent bundles pass through the fornix to NAc medium spiny neurons (French and Totterdell, 2002, 2003; Sesack and Grace, 2010; Britt et al., 2012; MacAskill et al., 2012).

The NAc works as an interface between the limbic cortex and the midbrain structures involved in motor performance. The fornix carries limbic inputs to the ventral striatum, which then projects them to the NAc. It also carries fibers arising in the septal area that project to the hippocampal formation and to other areas of the rostral forebrain (Boeijinga et al., 1993). Another study suggests that there may be indirect connections between the fornix and the NAc from the dorsal CA3 (*Cornu Ammonis* areas) via the VTA (Luo et al., 2011). A non-human primate study found that the fornix contains 500,000 fibers projecting to and from various regions, including projections from the CA3 that target the mammillary bodies and the NAc (Talakoub et al., 2016). An fMRI study previously reported that fornix DBS could serve as a functional connection between the medial limbic and mesocorticolimbic circuits and may modulate presynaptic dopamine efflux in the NAc (Ross et al., 2016). The combination of these anatomic and functional studies suggests that fornix stimulation drives NAc input and output, which triggers the activity of the hippocampus and/or the thalamus.

Several research papers have verified the neuronal framework for dopamine efflux in the NAc induced by fornix stimulation. One rodent study suggested that stimulation of the ventral subiculum of the hippocampus evoked dopamine release in the NAc by synaptic activation of both ionotropic and metabotropic glutamate receptors (Blaha et al., 1997). The majority of cells in the NAc are GABAergic neurons with predominantly extrinsic innervation via excitatory glutamatergic projections from the hippocampus, PFC, and AM (Kelley and Domesick, 1982; Friedman et al., 2002; Sesack and Grace, 2010). NAc dopamine is known to play an important role in motor activity and in behaviors governed by drugs and natural reinforcers, as well as in non-associative forms of learning (Mele et al., 2004). Because the fornix is part of the Papez circuit, fornix stimulation-driven efflux of dopamine in the NAc may carry information related to memory and emotion (Halbig et al., 2011). Previously, it was shown that synchronized electrical stimulation of the dopamine pathway and the hippocampal pathway generates an additive fMRI response in the NAc, suggesting a modulatory role for dopamine in the hippocampal pathway (Krautwald et al., 2013). By modulating hippocampal activity, dopamine is thought to play a role in the motivational relevance of memory content (Shohamy and Adcock, 2010).

We do not have a good explanation for the decreased metabolism in the PMC, PSC, and PVC. All of the positive metabolism brain areas correspond well with the Papez circuit, but these decreased-metabolism brain areas are not in the Papez circuit. First, looking for a possible circuitry connection and not discussing the decreased signal, one possible explanation of the effect in the PMC, PSC, and PVC is the role of the AM. The amygdaloid complex contains many nuclei involved in both sensory and motor functions (Vann and Nelson, 2015). The NAc and septal area also have sensory-motor connections, so there could be a possible secondary connection. This could be further supported by the NAc having a bilateral connection to both hemispheres. The NAc also has connections from the AM, supporting our finding of metabolic changes in the AM and neurochemical changes in the NAc after fornix stimulation. Our previous study (Ross et al., 2016) showed both ipsilateral and contralateral somatosensory BOLD responses, which indicated circuit involvement between the hippocampus and PSC. In terms of the functional distribution of the forniceal fibers, part of the fornix carries fibers from the caudal hippocampus that process exteroceptive signals (Raslau et al., 2015), and lesion or damage to the fornix lead to visual discrimination deficits (Lech et al., 2016). Another possible explanation of the decreases is that electrical stimulation affected not only the fornix but also brain regions near the fornix such as the hypothalamic area. Hypothalamic DBS studies reported that functional imaging revealed stimulation-induced deactivations in the PSC (May et al., 2006; May, 2008). The final possibility is that the observed changes are artifacts of the PET analysis method, for example, the proportional scaling for global normalization. This method would work well for focal changes induced by experimental treatments but could bias the statistical analysis when relatively wide brain areas are involved. Because local changes can be smeared in wide brain areas due to the low spatial resolution of [F-18]FDG micro-PET in rat neuroimaging, the proportional scaling may cause type I or type II errors in the analysis due to over- or under-estimation of global activity.

Among the limitations of the present study is the fact that the evoked dopamine findings represent the effects of short-term stimulation (2 s) due to the inherent long-term drifting of FSCV for longer periods (Heien et al., 2005), while FDG-PET can measure 30 min of continuous stimulation effects. Further study is needed to understand if these phasic dopamine findings would impact behavioral memory test scores. Secondly, experiments, such as this one, conducted under anesthesia may not represent the activation effects found when subjects are conscious. The micro-PET FDG protocol can be used to measure conscious-state brain activity (Mizuma et al., 2010), and thus this study likely confirms a platform from which to conduct conscious-state behavioral tests during FDG uptake and PET imaging. Lastly, an inherent limitation of electrical stimulation is that it can activate unwanted and non-specific brain areas near the targeted region. For this reason, we took a cautious approach to avoided possible direct stimulation of the mfb, which contains dopaminergic fibers, given that the electrical current spread for rodent-use micro-electrodes is reported to be <1 mm (Lozano et al., 2002). Although there are inherent limitations to translating findings from healthy small animals to human pathologic conditions, the global and local patterns of molecular imaging in this study reveal potential neuronal mechanisms underlying fornix DBS (Ross et al., 2016; Fu et al., 2018).

## Conclusion

In conclusion, the results of this rodent study provide a platform to investigate the interactions between the Papez and mesolimbic circuits related to certain aspects of memory function. Our findings support the concept that electrical stimulation of the fornix increases brain activity and controls dopamine efflux in the NAc and suggests that further exploration of the neuromodulatory effects of fornix DBS is warranted relative to its potential therapeutic impact on certain aspects of memory and emotional processing.

## Ethics Statement

All procedures were performed in accordance with the National Institutes of Health Guidelines for Animal Research (Guide for the Care and Use of Laboratory Animals), and the Hanyang University Institutional Animal Care and Use Committee approved all experimental procedures.

## Author Contributions

H-KM and DJ supervised all aspects of this work equally. HS, IK, KL, DJ, and H-KM designed the analyses. HS and H-KM conducted the analyses. HS, S-YL, H-UC, and YO collected the data. S-YL, KL, and DJ provided the resources and consultation on the section “Materials and Methods.” HS, DJ, and H-KM wrote the manuscript. All authors commented on and accepted the final version of the manuscript.

## Conflict of Interest

The authors declare that the research was conducted in the absence of any commercial or financial relationships that could be construed as a potential conflict of interest.

## Supplementary Material

FIGURE S1 Electrical stimulation evoked dopamine response changes by stimulation frequency and amplitude. **(A)** Dopamine oxidation current versus stimulation frequency from 10Hz to 200Hz. **(B)** Dopamine oxidation versus stimulation amplitude from 50 μA to 400 μA.
