# Supplementary material for: Fornix Stimulation Induces Metabolic Activity and Dopaminergic Response in the Nucleus Accumbens
Source: Front Neurosci. 2019 Oct 24;13:1109. doi: 10.3389/fnins.2019.01109 (PMC6821687; doi:10.3389/fnins.2019.01109)
Supplement: FIGURE S1 — Electrical stimulation evoked dopamine response changes by stimulation frequency and amplitude. (A) Dopamine oxidation current versus stimulation frequency from 10Hz to 200Hz. (B) Dopamine oxidation versus stimulation amplitude from 50 μA to 400 μA. [file Data_Sheet_1.PDF]

Supplementary Material

1 Supplementary Figures

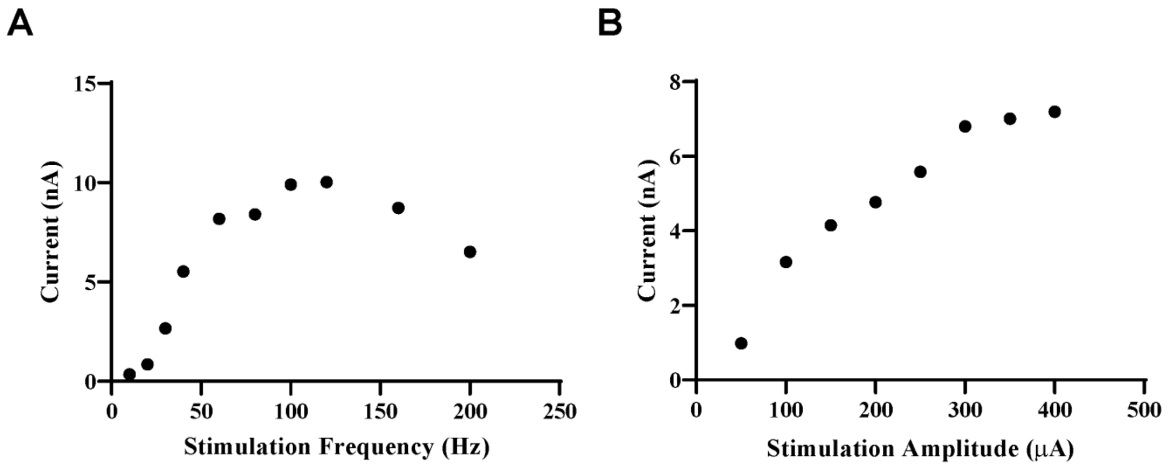

Supplementary Figure 1. (A) Dopamine oxidation current versus stimulation frequency from 10Hz to 200Hz. (B) Dopamine oxidation versus stimulation amplitude from 50μA to 400μA.

We applied electrical stimulation with varied frequency from 10Hz to 200Hz every 10 minutes (n=1). The highest dopamine response showed around 100 ~ 130Hz. Based on the result, we chose 120Hz for stimulation.

Measuring stimulation-induced dopamine under anesthesia state, both 100 μA vs 300 μA had good response of dopamine with difference in the release amount. In order to see clearer DA responses in FSCV 2sec stimulation, we used 300 μA stimulation (no induced motion under anesthesia state). For the awake 30min stimulation protocol in PET, we did saw some stimulation induced motion in the animal with 300 μA, so we had to reduce the stimulation amplitude down to 100 μA. There was no behavioral change of the awake rat during continuous stimulation using 100μA.
